# Detection of Prohibited Fish Drugs Using Silver Nanowires as Substrate for Surface-Enhanced Raman Scattering

**DOI:** 10.3390/nano6090175

**Published:** 2016-09-21

**Authors:** Jia Song, Yiqun Huang, Yuxia Fan, Zhihui Zhao, Wansong Yu, Barbara A. Rasco, Keqiang Lai

**Affiliations:** 1 College of Food Science and Technology, Shanghai Ocean University, Shanghai 201306, China; susanjia123@163.com (J.S.); yiqunh@hotmail.com (Y.H.); yxfan@shou.edu.cn (Y.F.); zhihuiz2015@sohu.com (Z.Z.); dasong1990@126.com (W.Y.); 2 Engineering Research Center of Food Thermal-processing Technology, Shanghai Ocean University, Shanghai 201306, China; 3 School of Food Science, Washington State University, Pullman, WA 99165, USA; rasco@wsu.edu

**Keywords:** SERS, silver nanowires, glycerol, malachite green, crystal violet, furazolidone, chloramphenicol

## Abstract

Surface-enhanced Raman scattering or surface-enhanced Raman spectroscopy (SERS) is a promising detection technology, and has captured increasing attention. Silver nanowires were synthesized using a rapid polyol method and optimized through adjustment of the molar ratio of poly(vinyl pyrrolidone) and silver nitrate in a glycerol system. Ultraviolet-visible spectrometry, X-ray diffraction, and transmission electron microscopy were used to characterize the silver nanowires. The optimal silver nanowires were used as a SERS substrate to detect prohibited fish drugs, including malachite green, crystal violet, furazolidone, and chloramphenicol. The SERS spectra of crystal violet could be clearly identified at concentrations as low as 0.01 ng/mL. The minimum detectable concentration for malachite green was 0.05 ng/mL, and for both furazolidone and chloramphenicol were 0.1 μg/mL. The results showed that the as-prepared Ag nanowires SERS substrate exhibits high sensitivity and activity.

## 1. Introduction

Surface-enhanced Raman scattering or surface-enhanced Raman spectroscopy (SERS) has served as an effective tool to detect molecules or biomolecules adsorbed onto the surface of metallic nanomaterials [1]. SERS technology has been applied in various fields, including materials science, bio-sensing, environmental science, and food science [2]. It is well known that the successful application of SERS is strongly determined by the composition and surface morphology of metallic nanostructures. The recent dramatic development of nanomaterials is the driving force for the wide applications of SERS technology [3].

Silver nanomaterials have been intensively studied and extensively applied in various fields, due to their special dielectric property leading to strong plasmon absorption in the visible region [4]. One-dimensional (1D) silver nanoparticles with different shapes and sizes, such as wires, rods, tubes, and belts have been synthesized, and each of them has shown some unique optical, thermal, or electrical properties [5,6]. Silver nanowires (Ag NWs) with well-controlled dimension and finite length are valuable plasmonic structures, on account of the tremendous enhancement of polarizability and appropriate waveguides in virtue of the transmission light across the (sub)micrometer length scale [7,8]. Thanks to their large surface areas, coupled with their electromagnetic properties, Ag NWs have been successfully applied as a SERS substrate for the analysis of Rhodamine 6G, 2,4-dinitrotoluene, pyridine, and so on [9,10].

Among various approaches developed for the fabrication of Ag NWs, the polyol process is one of the most promising methods in terms of cost, yield, and simplicity [11]. Typically, the Ag NWs are synthetized by the reduction of a silver precursor at elevated temperature with the cooperation of a stabilizing agent; polyol is usually used as both solvent and reductant in the method, silver nitrate (AgNO_3_) as the silver precursor, and poly(vinyl pyrrolidone) (PVP) as the stabilizing agent or surfactant. Because of its non-toxicity, high boiling point (290 °C), and low-cost, glycerol is considered as a kind of favorable polyol in the synthetic reaction [12].

The purpose of this study was to prepare Ag NWs via the polyol process and to apply them as a SERS substrate for the detection of trace levels of prohibited fish drugs, including malachite green (MG), crystal violet (CV), furazolidone, and chloramphenicol. Although these drugs are highly effective against fish diseases caused by fungal and bacterial infections in aquatic environments, they are banned in aquaculture practices because they may pose health risks and adverse environmental impact [13]. However, these prohibited drugs are still frequently used in many places around the world, due to their low cost and effectiveness in the control of fish diseases [14,15,16]. Ultra-sensitive chromatographic methods, such as liquid chromatography-tandem mass spectrometry (LC-MS/MS) with a limit of detection equal to or below 1 ppb are required to analyze these zero-tolerance chemicals in fish and fish products [17]. SERS methods could be sensitive, easy-to-use, and affordable, serving as a good alternative to the more complicated and expensive chromatographic method. Our group’s previous studies involving the use of commercial SERS substrates and lab-synthesized Au nanospheres as SERS substrates led to less ideal results in sensitivity: the lowest detectible concentrations for standard solutions were 0.5–700 ppb for MG, 0.5–50 ppb for CV, and 0.8–5 ppm for furazolidone [18,19,20,21,22]. This study is a continuation of our previous work on the development of more sensitive SERS approaches for the detection of banned fish drugs. The optimal silver nanowires were synthesized by examining the influence of the molar ratio of PVP to AgNO_3_ on the morphology of nanoparticles in a glycerol system, which was seldom reported. SERS sensitivity of the as-prepared Ag NWs was subsequently evaluated by using them as the SERS substrate for the detection of MG, CV, furazolidone, and chloramphenicol. Based on this study, Ag NWs with high quality, high yield, and high aspect ratios were synthesized in an easy-to-operate, time-efficient, low-cost way, and the as-prepared substrate combined with SERS technology shows a promising future for the ultra-sensitive determination of the banned chemicals in foodstuffs.

## 2. Results and Discussion

### 2.1. The Effect of Molar Ratio of PVP and AgNO_3_ in the Synthesis of Ag NWs

**Figure 1:**
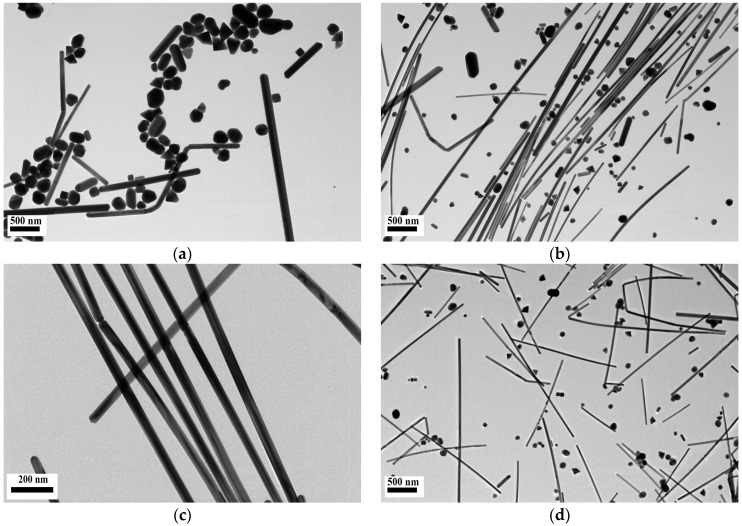
Transmission electron microscopy (TEM) images of Ag nanomaterials obtained through the use of different molar ratios of poly(vinyl pyrrolidone) (PVP) to silver nitrate (AgNO_3_): (**a**) 0.5:1, (**b**) 3:1, (**c**) 5.5:1, (**d**) 8:1.

Figure 1 shows the transmission electron microscopy (TEM) images of silver nanomaterials synthetized using different molar ratios of PVP to AgNO_3_ ranging from 0.5 to 8, indicating that the final morphology of silver nanomaterials synthesized through the polyol method is highly dependent upon the molar ratio between PVP and AgNO_3_.

When the molar ratio of PVP to AgNO_3_ was 0.5, highly irregular nanoparticles, including nanospheres, nanocubes, nanorods, and short nanowires were formed (Figure 1a). As the molar ratio increased to three, there was a significant increase in the number of Ag NWs, but other sizes or shapes of nanoparticles were still present (Figure 1b). When the molar ratio increased to 5.5, Ag NWs with high productivity and aspect ratios were synthesized (Figure 1c). A further increase in the molar ratio led to the formation of nanowires with the smallest diameter and other shapes of nanoparticles, such as nanorods and nanospheres (Figure 1d). This phenomenon can be explained by the fact that PVP may interact more strongly with the {100} facets (the lateral surfaces) than with the {111} facets (the end surfaces), due to selective adsorption; under a suitable molar ratio of PVP and AgNO_3_, {100} facets are passivated by the adsorption of PVP, and the silver atoms are deposited at the reactive {111} facets for 1D-preferential growth. However, excessive PVP could lead to the hindrance of the anisotropic growth of 1D nanomaterials and the appearance of small-sized particles, as all facets are covered and the Ag seed surface loses selectivity. On the other hand, when the molar ratio was relatively low (less than three), some silver seeds grew into irregular shapes and sizes [23,24].

Table 1 provides a summary of the diameters and lengths of the as-obtained silver nanoparticles. The results indicate that with the increase of the molar ratio between PVP and AgNO_3_, the dimeter of the as-obtained Ag NWs continuously decreased from 142.5 ± 58.3 nm to 35.4 ± 9.8 nm, while the length increased from 0.3–3 μm to 7–10 μm and then decreased to 0.7–5 μm. Based on the relative standard deviation (RSD) value for the diameters of the nanowires, when the molar ratio of PVP to AgNO_3_ was 5.5, nanowires with relatively uniform shapes were formed, which could very likely provide relatively-high reproducibility of enhanced Raman scattering signals.

### 2.2. Characterization of Ag NWs

Silver nanoparticles with different shapes and sizes exhibit different surface plasmon resonance (SPR) bands located at different frequencies. The ultraviolet-visible (UV–Vis) spectrometer is a useful tool to investigate the SPR properties [25].

**Figure 2:**
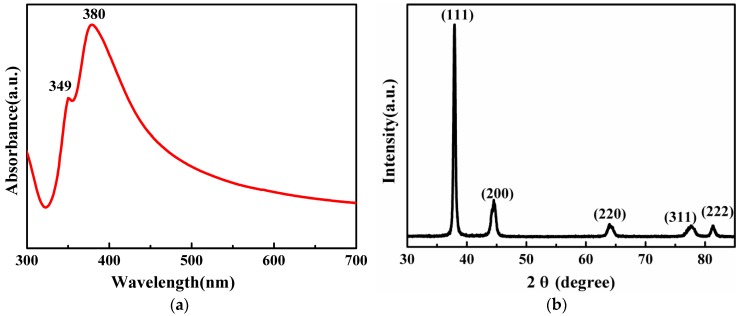
(**a**) Ultraviolet-visible (UV-Vis) absorbance spectrum of silver nanowires; (**b**) X-ray diffraction (XRD) pattern of silver nanowires.

Unlike silver nanospheres, the UV-Vis absorbance spectrum of the 49.4 ± 3.9 nm diameter Ag NWs (Figure 1c) exhibits two characteristic absorption peaks at 349 nm and 380 nm, as shown in Figure 2a. The two characteristic peaks were related to transversal resonance modes with a cross-section of pentagonal shapes for 1D silver nanoparticles. The peak at 349 nm was ascribed to the out-of-plane quadrupole resonance, while the stronger peak at 380 nm could be attributed to the out-of-plane dipole resonance of Ag NWs. In addition, the longitudinal mode of the as-prepared Ag NWs disappeared, probably because of their relatively high aspect ratios [26].

The X-ray diffraction (XRD) pattern of the as-prepared Ag NWs is shown in Figure 2b, and five characteristic diffraction peaks were observed. The positions of the characteristic diffraction peaks at 2θ of 38.1°, 44.3°, 64.4°, 77.4°, and 81.5° corresponded to (111), (200), (220), (311), and (222) planes of face-centered cubic (FCC) silver; the lattice constant calculated from this pattern was 4.0862 Å, which is identified well with the standard PDF card (a = b = c = 4.0862 Å, JCPDS File No. 04-0783) [27]. The high intensity ratio between (111) and (200) peaks indicates the abundance of (111) crystalline planes for the Ag NWs [28]. Moreover, there were no other impurity diffraction peaks in Figure 2b, suggesting the high purity of the resulting product.

### 2.3. SERS Analysis of Malachite Green, Crystal Violet, Furazolidone, and Chloramphenicol

**Figure 3:**
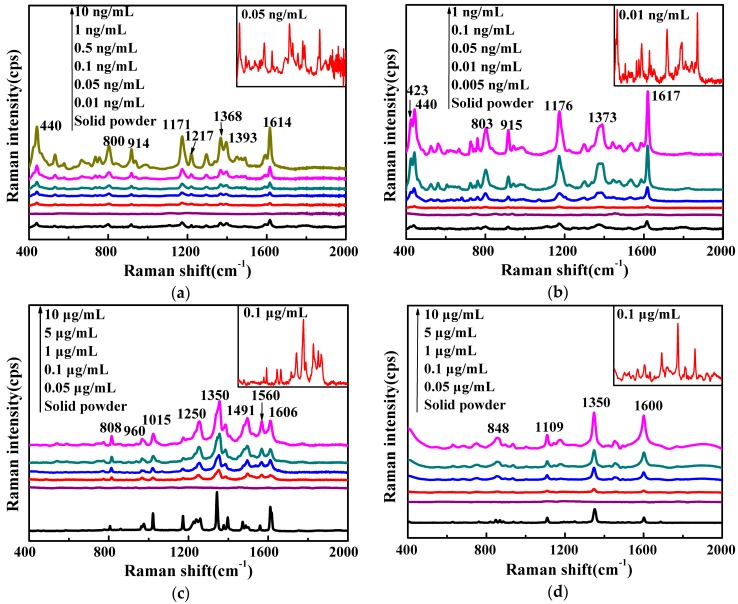
Raman and surface-enhanced Raman scattering (SERS) optical spectra of (**a**) malachite green, (**b**) crystal violet, (**c**) furazolidone, (**d**) chloramphenicol.

The as-prepared Ag NWs were used as a SERS substrate for the detection of banned fish drugs, including MG, CV, furazolidone, and chloramphenicol. Figure 3 shows the Raman spectral features of the solid powder of each drug and SERS spectra of their standard solutions, and Table 2 shows the assignments of their vibrational bands.

Both MG and CV are triphenylmethane dyes with highly effective anti-fungal properties, and are banned in many countries due to potential carcinogenesis, teratogenesis, and mutagenesis in human beings [29]. However, MG and CV are illegally used in aquaculture because of their low cost, ease of use, and high effectiveness. Particularly for MG, it was the number one illegal drug found in fish and fish products in the past ten years, based upon the database of the Rapid Alert System for Food and Feed of European Union [30]. In our previous study, we were able to detect MG and CV at concentrations as low as 0.1 ng/mL using Au–Ag core–shell (Au@Ag) bimetallic nanoparticles [31], which is much more sensitive than that using two commercial SERS substrates and as-obtained Au nanospheres [18,19,20,21,22]. However, because of the effect of sample matrices, the sensitivity for the detection of MG in actual fish muscle could be decreased by as much as 20 times compared to that in standard solutions [22]. Therefore, the substrates used in our previous studies could not completely satisfy the requirement for the limit of detection of 1 ppb or below. As shown in Figure 3a, the prominent characteristic bands for MG at 440 cm^−1^, 1171 cm^−1^, 1368 cm^−1^ and 1614 cm^−1^ are attributed to out-of-plane phenyl–C–phenyl bending, in-plane C–H bending, in-plane N–phenyl stretching vibration, and in-plane C–C stretching vibration of the ring [32]. Based upon the SERS spectra, MG could be clearly identified at levels as low as 0.05 ng/mL (1.08 × 10^−10^ M), which approaches the concentration (1.0 × 10^−10^ M) in the reported study [33]. Comparing Figure 3b with Figure 3a, the Raman optical spectrum of CV solid and SERS optical spectra of CV standard solutions were similar to MG, due to their similar chemical structures. The characteristic peaks of CV at 1617 cm^−1^ (in-plane C–C stretching vibration of the ring), 1373 cm^−1^ (overlapping effect of in-plane N–phenyl stretching vibration and in-plane C–C stretching vibration), 1176 cm^−1^ (in-plane aromatic C–H vibration), 915 cm^−1^ (ring-breathing), 803 cm^−1^ (out-of-plane phenyl–H bending), and 423 cm^−1^ and 440 cm^−1^ (out-of-plane phenyl–C–phenyl bending) could be identified at concentrations as low as 0.01 ng/mL (2.45 × 10^−11^ M) [34]. This indicates that the as-prepared Ag NWs have great advantages in terms of SERS sensitivity for MG and CV, compared to the two commercial substrates, Au nanospheres and Au@Ag bimetallic nanospheres synthesized in our lab and used in our previous studies. The sensitivity very likely could meet the general legal requirements for the sensitivity (limit of detection of 1 ppm or below) of an analytical method for the detection of prohibited fish drugs in aquatic food products.

Furazolidone is a nitrofuran antibacterial drug that has been prohibited in China, the USA, and the European Union because of the potential genotoxic and carcinogenic effects to human bodies [35]. The as-prepared Ag NWs were also used as a SERS substrate to acquire the spectra of furazolidone at different concentrations. As shown in Figure 3c, the peaks at 1015 cm^−1^, 1350 cm^−1^, and 1491 cm^−1^ are attributed to stretching vibrations of the furan ring. The other characteristic peak at 1560 cm^−1^ is assigned to the asymmetric stretching of NO_2_ and symmetric stretching of the furan ring. The bands at 808 cm^−1^ and 960 cm^−1^ are related to the out-of-plane bending of C–H and the symmetric stretching of C–C, respectively [36]. The characteristic peaks of furazolidone were clearly visible at concentrations as low as 0.1 μg/mL.

Chloramphenicol possesses broad-spectrum antibiotic properties and is used in aquaculture practices to prevent and control some fish diseases. The residual chloramphenicol in food could cause bone marrow suppression and aplastic anemia; therefore, the use of chloramphenicol in aquaculture is banned by many countries, including the USA, Japan, China, and the European Union [38]. In addition, the exposure to antibiotics may give rise to antibacterial resistance in human beings. Figure 3d shows the SERS spectra of chloramphenicol solutions (0.05–10 μg/mL) collected with the Ag NWs as substrate. The prominent brands at 848 cm^−1^, 1109 cm^−1^, 1350 cm^−1^, and 1600 cm^−1^ are attributed to ring-breathing, in-plane bending of N–H, symmetric stretching of NO_2_, and ring stretching vibration for chloramphenicol [37]. The four characteristic peaks at around 848 cm^−1^, 1109 cm^−1^, 1350 cm^−1^, and 1600 cm^−1^ could be discernable in the SERS spectra of chloramphenicol solutions at concentrations as low as 0.1 μg/mL.

The use of the as-prepared Ag NWs as a SERS substrate could provide significant enhancement effects for the Raman scattering signals of banned fish drugs, particularly for CV and MG. Based upon the method described in Ru et al. [39], the enhancement factor for CV was calculated as 4.7 × 10^7^ (based upon the strongest peak at 1617 cm^−1^). As to the reproducibility of Ag NWs as a SERS substrate, the SERS spectra of 0.1 ng/mL CV standard solution collected with the as-prepared Ag NWs from 10 different batches synthesized in different days were quite consistent. The statistical RSD values of Raman intensity at 440 cm^−1^, 1176 cm^−1^, and 1617 cm^−1^ were calculated as 14.1%, 11.2%, and 9.8%, respectively, indicating the relatively high reproducibility of the as-prepared Ag NWs as a SERS substrate.

## 3. Materials and Methods

### 3.1. Materials

AgNO_3_ (>99%), PVP (M_w_ = 55,000 g/mol), sodium chloride (NaCl, ≥99.999%), CV (≥90%), MG (ACS grade), and furazolidone (ACS grade) were purchased from Sigma-Aldrich (St. Louis, MO, USA). Chloramphenicol (>99%) was purchased from J&K Co., Ltd. (Shanghai, China). Glycerol (ACS grade) was obtained from Aladdin Industrial Corporation. Ethanol (analytical grade), acetonitrile (HPLC reagent), and methanol (HPLC reagent) were from Sinopharm Chemical Reagent Co., Ltd. (Shanghai, China). Ultrapure water (18.2 MΩ·cm) was obtained with a Simplicity UV purification system (Millipore Corp., Billerica, MA, USA) and used throughout the experimental procedure. All glass apparatuses used were soaked with aqua regia for more than two hours and then washed three times with ultrapure water.

### 3.2. Synthesis of Ag NWs

Ag NWs were fabricated by a modified Yang’s method [40]. In brief, the mixture of PVP powder (0.516–8.250 g) and 190 mL glycerol in a three-necked round-bottomed flask was gradually heated up to 80 °C with gentle stirring until a transparent solution was formed. After being naturally cooled down to room temperature, 1.58 g of AgNO_3_ was added into the solution. Then, the solution was stirred vigorously to obtain a homogeneous solution. The mixture of salt solution (0.0585 g NaCl dissolved in 0.5 mL water) and 10 mL glycerol was added into the flask and heated to 210 °C in 20 min with slight stirring. Following this, the mixture was cooled down to room temperature, diluted with water by a volume ratio of 1:1, and then centrifuged at 8000 rpm for 15 min. The supernatant was cautiously discarded, and the residual precipitate was further washed three times by ethanol aqueous solution to remove the PVP residue and other impurities. The final product was dispersed in water and stored in a dark environment at refrigerator temperature (0–4 °C) within one week before being used as a SERS substrate.

### 3.3. Characterization of Ag NWs

The optical absorption spectrum (300 to 700 nm) of Ag NWs was was performed on a UV-Vis spectrometer (UV3000PC, MAPADA Instruments Ltd., Shanghai, China) in a quartz cuvette with an optical path of 1 cm. The crystal structures and phase composition of samples were investigated by X-ray diffraction (XRD, D/max-2600PC, Rigaku Ltd., Tokyo, Japan) with Cu-Kα radiation. The size and surface morphology of Ag nanoparticles were analyzed with transmission electron microscopy (TEM, JEM-2100F, JEOL Ltd., Tokyo, Japan). The average diameters and lengths of nanowires or nanorods were calculated based on 100 particles according to their TEM images.

### 3.4. Preparation of Standard Solution

Acetonitrile was used as solvent for MG, CV, and furazolidone standard solutions ranging from 0.005 to 10 ng/mL. Chloramphenicol was dissolved in methanol to prepare a series of standard solutions of 0.05, 0.1, 1, 5, and 10 μg/mL.

### 3.5. Raman and SERS Measurement

A Nicolet DXR microscopy Raman spectrometer (Thermo Fisher Scientific Inc., Waltham, MA, USA) equipped with a 633 nm He-Ne laser source was used in our study. A 20× microscope objective and 5 mW laser power were used for the acquisition of the normal Raman and SERS spectra (400–2000 cm^−1^). The selection of a 633 nm laser source was based upon the results of our previous studies on CV and MG [19,21,31]. The maximum absorption wavelength of CV (590 nm) and MG (620 nm) are close to the 633 nm laser excitation wavelength, leading to strong resonance enhancement and an overall high enhancement factor [22,31].

To obtain the normal Raman spectrum of a fish drug, a small amount of MG, CV, furazolidone, or chloramphenicol was deposited onto a glass slide and squeezed to a thin film, and then their normal Raman spectra were recorded. For SERS spectra collection, the as-prepared silver colloidal sol was mixed with standard solution and then vortexed for 10 s. Following this, 5 μL of the mixture was pipetted onto a glass slide and SERS spectra were subsequently recorded after the solvent was evaporated completely. Each SERS spectrum was an average of 20 spectra collected from 10 different random locations on each of two batches of as-prepared Ag NWs. To ensure the reproducibility of SERS spectra collected with substrates from different batches, SERS spectra of 0.1 ng/mL CV were acquired before using a new batch of substrate for the collection of SERS spectra of any fish drugs.

## 4. Conclusions

Ag NWs with high quality, high yield, and high aspect ratios were synthesized by the reduction of silver nitrate with glycerol in a simple, rapid, and economical approach; in addition, the solvents used were cost-efficient, environmentally-friendly, and non-toxic. The effects of the molar ratio of PVP to AgNO_3_ on the morphology of synthesized nanomaterials were investigated. The optimal silver nanowires were used as a SERS substrate to detect prohibited fish drugs, including malachite green, crystal violet, furazolidone, and chloramphenicol. The optimal Ag NWs coupled with SERS technology have extensive applications in the detection of illegal fish drugs. The minimum detectable concentrations for CV and MG were 0.01 ng/mL and 0.05 ng/mL, respectively, while for both furazolidone and chloramphenicol standard solutions were 0.1 μg/mL. The results showed that the as-prepared Ag nanowires SERS substrate exhibits high sensitivity and relatively good reproducibility for the analysis of prohibited fish drugs.

## Figures and Tables

**Table 1:** Mean diameter and length of the as-obtained silver nanowires or silver nanorods.

Molar ratio of PVP and AgNO_3_	Diameter (nm)	RSD (%) of Diameter	Length (μm)
0.5	142.5 ± 58.3	40.9	0.3–3
3	51.3 ± 20.4	39.9	6–10
5.5	49.4 ± 3.9	7.9	7–10
8	35.4 ± 9.8	27.8	0.7–5

RSD: Relative standard deviation.

**Table 2:** Assignments of vibrational bands of malachite green, crystal violet, furazolidone, and chloramphenicol [32,34,36,37].

Chemical Compounds	Raman Shift (cm^−1^)	Band Assignment
Malachite green	440	γ (phenyl–C–phenyl)
	800	γ (C–H)
	914	Ring-breathing
	1171	δ (C–H)
	1368	υ_ip_ (N–phenyl), υ_ip_ (C–C)
	1614	υ_ip_ (C–C)
Crystal violet	423, 440	γ (phenyl–C–phenyl)
	803	γ (C–H)
	915	Ring-breathing
	1176	υ_ip_ (C–H)
	1373	υ_ip_ (N–phenyl), υ_ip_ (C–C)
	1617	υ_ip_ (C–C)
Furazolidone	808	γ (C–H)
	960	υ (C–C)
	1015	υ (ring)
	1250	δ (C–H)
	1350	υ (ring)
	1491	υ_s,ip_ (ring)
	1560	υ_as_ (NO_2_), υ_s_ (ring)
	1606	υ_s,ip_ (C=N)
Chloramphenicol	848	Ring-breathing
	1109	δ (N–H)
	1350	υ_s_ (NO_2_)
	1600	Ring stretching

υ_s_: Symmetric stretching; υ_as_: Asymmetric stretching; δ: In-plane bending; γ: Out-of-plane bending; ip: In-plane.

## Abbreviations

The following abbreviations are used in this manuscript:

SERS	Surface-enhanced Raman scattering or surface-enhanced Raman spectroscopy
1D	One-dimensional
Ag NWs	Silver nanowires
PVP	Poly(vinyl pyrrolidone)
AgNO_3_	Silver nitrate
MG	Malachite green
CV	Crystal violet
LC-MS/MS	Liquid chromatography-tandem mass spectrometry
Au@Ag	Au–Ag core–shell
RSD	Relative standard deviation
UV-Vis	Ultraviolet-Visible
XRD	X-ray diffraction
TEM	Transmission electron microscopy
SPR	Surface plasmon resonance
FCC	Face-centered cubic
NaCl	Sodium chloride
